# Increased attenuation but decreased immunogenicity by deletion of multiple vaccinia virus immunomodulators

**DOI:** 10.1016/j.vaccine.2016.08.002

**Published:** 2016-09-14

**Authors:** Rebecca P. Sumner, Hongwei Ren, Brian J. Ferguson, Geoffrey L. Smith

**Affiliations:** Department of Pathology, University of Cambridge, Tennis Court Road, Cambridge CB2 1QP, UK

**Keywords:** Vaccinia virus, Immunogenicity, NF-κB inhibitor, IRF3 inhibitor, Vaccine efficacy

## Abstract

Vaccinia virus (VACV)-derived vectors are popular candidates for vaccination against diseases such as HIV-1, malaria and tuberculosis. However, their genomes encode a multitude of proteins with immunomodulatory functions, several of which reduce the immunogenicity of these vectors. Hitherto only limited studies have investigated whether the removal of these immunomodulatory genes in combination can increase vaccine efficacy further. To this end we constructed viruses based on VACV strain Western Reserve (WR) lacking up to three intracellular innate immunomodulators (N1, C6 and K7). These genes were selected because the encoded proteins had known functions in innate immunity and the deletion of each gene individually had caused a decrease in virus virulence in the murine intranasal and intradermal models of infection and an increase in immunogenicity. Data presented here demonstrate that deletion of two, or three of these genes in combination attenuated the virus further in an incremental manner. However, when vaccinated mice were challenged with VACV WR the double and triple gene deletion viruses provided weaker protection against challenge. This was accompanied by inferior memory CD8^+^ T cell responses and lower neutralising antibody titres. This study indicates that, at least for the three genes studied in the context of VACV WR, the single gene deletion viruses are the best vaccine vectors, and that increased attenuation induced by deletion of additional genes decreased immunogenicity. These data highlight the fine balance and complex relationship between viral attenuation and immunogenicity. Given that the proteins encoded by the genes examined in this study are known to affect specific aspects of innate immunity, the set of viruses constructed here are interesting tools to probe the role of the innate immune response in influencing immune memory and vaccine efficacy.

## Highlights

• Vaccinia virus-derived vaccine vectors are being engineered to improve immunogenicity.
• Deleting genes with immunomodulatory function can increase immunogenicity and decrease virulence.
• Deletion of *N1L*, *C6L* or *K7R* individually improves immunogenicity, but not in combination.
• A virus lacking all three genes induces poorer CD8^+^ T cell and neutralising antibody responses.

## 1 Introduction

The poxvirus family is a diverse group of dsDNA viruses that replicate in the cytoplasm of infected cells. The potential use of this family, particularly the well-characterised vaccinia virus (VACV) strains, as vaccine vectors has been under investigation since the 1980s [1]. VACV has many features that make it attractive for this use; a large capacity for foreign gene expression, relative ease of manipulation, and, importantly, induction of strong innate and memory immune responses, including both cellular and humoral arms [2]. Much research has focused on modified virus Ankara (MVA) and NY-VAC because of their severe attenuation, and, at least for MVA, the inability to replicate in most mammalian cells. These vectors are safe even in immunocompromised hosts [3], [4], which is of particular importance for vaccination against diseases such as HIV-1, malaria and tuberculosis [2]. There is still a need to improve the immunogenicity of these vectors, particularly because large viral doses, repeated vaccination or prime-boost regimes are required to achieve adequate correlates of protection [5].

The host antiviral innate immune response provides a strong selective pressure and, consequently, viruses have evolved a plethora of mechanisms to counteract its effects. Engagement of viral components with innate immune receptors activates transcription factors including nuclear factor kappa-light-chain-enhancer of activated B cells (NF-κB) and interferon (IFN) regulated factors (IRF)-3/7 that coordinate the production of pro-inflammatory cytokines, chemokines and type I IFNs (reviewed in [6], [7]). Importantly this pro-inflammatory milieu attracts professional antigen presenting cells to the site of infection, providing an important link to the adaptive immune response and the subsequent establishment of immune memory.

VACV dedicates between one third and one half of its genome to dampening host innate responses (reviewed in [6]). For example, nine intracellular NF-κB inhibitors have been identified and there is evidence that more remain to be discovered [8]. In addition, VACV encodes numerous IRF-3/7 inhibitors and multiple mechanisms to counteract the actions of IFNs [6]. The mechanisms by which innate immunity impacts successful vaccine design remain incompletely understood. Importantly, recent data have demonstrated that the deletion of several VACV immunomodulatory genes individually enhances the immunogenicity of these vectors (reviewed in [6], [9]). Examples of such genes include the NF-κB inhibitor N1 [10], the IRF-3/7 inhibitor C6 [11], [12] and the dual NF-κB and IRF-3/7 inhibitor K7 ([13] and Ren et al., unpublished data). Proteins N1 and K7 have Bcl-2 folds [14], [15] and C6 is predicted to do so [16] and all are virulence factors [17], [18], [19].

Some studies have investigated the effect of deleting multiple immunomodulatory genes from VACV vectors but so far have not included an in-depth comparison of single gene deletions in isolation versus deletion of genes in combination [13], [20], [21]. These comparisons have also not always included challenge experiments, instead measuring aspects of immunological memory that may correlate with immune protection. This study therefore tested whether the immunogenicity of VACV could be improved further by deleting three intracellular innate immunomodulators in combination (encoded by genes *N1L*, *C6L* and *K7R*) from VACV strain Western Reserve (WR). These immunomodulators were selected because their function in innate immunity was known, and because deletion of each of these genes in isolation from VACV WR increased immunogenicity, but decreased virulence. This study shows that deletion of these three genes did not affect VACV growth *in vitro*, but led to sequential attenuation of the virus in two *in vivo* models. In challenge models, despite each individual gene deletion enhancing immunogenicity, a virus lacking all three genes was a poorer vaccine, accompanied by inferior memory T cell responses and lower neutralising antibody titres. This illustrates how the design of vaccines for optimal immunogenicity must consider how the degree of attenuation impacts on the induction of immunological memory.

## 2 Materials and methods

### 2.1 Cells, viruses and mice

BSC-1 and CV-1 cells were grown in Dulbecco’s Modified Eagle’s Medium (DMEM, GIBCO) supplemented with 10% (v/v) foetal bovine serum (FBS) (Sigma-Aldrich, St. Louis, USA) and penicillin/streptomycin (P/S, 50 μg/ml, Gibco, NY, USA). EL-4 cells were grown in Roswell Park Memorial Institute medium (RPMI, GIBCO, Paisley, UK) supplemented with 10% (v/v) FBS and 50 μg/ml P/S. The deletion and revertant VACVs for *N1L* [17], *C6L* [18] and *K7R* [19] were described previously. Female mice Balb/c and C57/B6 (6–8 weeks old) were purchased from Harlan (Blackthorn, United Kingdom).

### 2.2 Construction of Western Reserve recombinant viruses

Viruses were constructed using the transient dominant selection method [22] as described [18], using vΔN1 [17] as a starting point and plasmids Z11ΔC6 [18] and pSJH7-ΔK7 [19] to delete *C6L* and *K7R* respectively. Z11 is a pCI-derived plasmid containing the *Escherichia coli guanylphosphoribosyl transferase* (*Ecogpt*) gene fused in-frame with the *enhanced green fluorescent protein* (*EGFP*) gene under the control of the VACV 7.5 K promoter. Revertant viruses were constructed by replacing the deleted genes in their natural loci using plasmids Z11C6Rev [18] and pSJH7-K7 [19]. The genotype of resolved viruses was analysed by PCR following proteinase K-treatment of infected BSC-1 cells using primers that anneal to the flanking regions of *N1L*, *C6L* and *K7R* [17], [18], [19]. Infectious virus titres were determined by plaque assay on BSC-1 cells.

### 2.3 SDS-PAGE and immunoblotting

Infected BSC-1 cells were lysed in a cell lysis buffer containing 50 mM Tris pH 8, 150 mM NaCl, 1 mM EDTA, 10% (v/v) glycerol, 1% (v/v) Triton X100, 0.05% (v/v) NP40 supplemented with protease inhibitors (Roche). Samples were boiled for 5 min and then subjected to SDS-PAGE. Primary antibodies were from the following sources: mouse anti-α-tubulin (Upstate Biotech), mouse anti-D8 mAb AB1.1 [23], rabbit-anti-N1 polyclonal antiserum [17], rabbit-anti-C6 polyclonal antiserum [18] and rabbit-anti-K7 polyclonal antiserum [24]. Primary antibodies were detected with goat-anti-mouse/rabbit IRdye 800CW infrared dye secondary antibodies and membranes were imaged using an Odyssey Infrared Imager (LI-COR Biosciences).

### 2.4 Plaque size analysis

BSC-1 cells were inoculated at approximately 50 plaque forming units (p.f.u.) per well of a 6-well plate and stained with crystal violet 3 days later [18]. The sizes of 20 plaques per well were measured using Axiovision acquisition software and a Zeiss AxioVert.A1 inverted microscope as described [25].

### 2.5 Murine intranasal and intradermal models of infection

Female BALB/c mice (*n* = 5, 6–8 weeks old) were infected intranasally (i.n.) with 5 × 10^3^ p.f.u. of purified VACV strains. VACV was purified from cytoplasmic extracts of infected cells by two rounds of sedimentation through 36% (w/v) sucrose at 32,900*g* for 80 min. Virus was resuspended in 10 mM Tris-HCl pH 9. Virus used for infections was diluted in phosphate-buffered saline containing 1% bovine serum albumin and the titre of the diluted virus that was used to infect mice was determined by plaque assay on the day of infection. Mice were monitored daily to record body weight and signs of illness as described [26], [27]. Female C57BL/6 mice (*n* = 5, 6–8 weeks old) were inoculated intradermally (i.d.) in both ear pinnae with 10^4^ p.f.u. and the resulting lesions were measured daily as described [28], [29]. For the challenge experiments, mice that had been inoculated i.n. were challenged 6 weeks later and mice that had been inoculated i.d. were challenged 4 weeks later, i.n., with 5 × 10^6^ p.f.u. of wild-type VACV WR.

### 2.6 Intracellular cytokine staining

Splenocytes were prepared as described [10] and incubated for 4 h with a C57BL/6-specific CD8^+^ VACV peptide B8_20–27_ [30] or a negative control CD8^+^ VACV peptide specific for BALB/c mice, E3_140–148_ [31] at a final concentration of 0.1 μg/ml. After 1 h Golgi stop (BD Biosciences) was added and the cells were incubated for a further 3 h. Cells were then stained for CD8 and either IFNγ or TNFα and analysed by flow cytometry [10].

### 2.7 ^51^Cr release cytotoxic assay

Cytotoxic T lymphocyte (CTL) activity was assayed with a standard ^51^Cr-release assay using VACV-infected EL-4 cells as targets, as described [32]. The percentage of specific ^51^Cr-release was calculated as specific lysis = [(experimental release − spontaneous release)/(total detergent release − spontaneous release)] × 100. The spontaneous release values were always <5% of total lysis.

### 2.8 IMV plaque reduction neutralisation assay

The neutralising titre of anti-VACV antibodies was calculated by plaque assay on BSC-1 cells as described [10]. Neutralisation dose 50 (ND_50_) values represent the reciprocal of the serum dilution giving 50% reduction in plaque number compared with virus incubated without serum.

### 2.9 Serum antibody titration by ELISA

The binding of serum antibodies to VACV-specific epitopes was measured by enzyme-linked immunosorbent assay (ELISA) using plates coated with lysates of VACV strain WR-infected cells that had been treated with ultraviolet light and psoralen to inactivate VACV infectivity as described [33], [34]. Plates coated with bovine serum albumin were used as a negative control. IgG end-point titres were defined as the reciprocal serum dilutions giving twice the average optical density values obtained with bovine serum albumin.

### 2.10 Statistical analysis

Data were analysed using an un-paired Student’s *t*-test, with Welch’s correction where appropriate, or a Mann–Whitney test as indicated. Statistical significance is expressed as follows: ^*^P < 0.05, ^**^P < 0.01, ^***^P < 0.001.

### 2.11 Ethics statement

This work was carried out in accordance with regulations of The Animals (Scientific Procedures) Act 1986. All procedures were approved by the United Kingdom Home Office and carried out under the Home Office project licence PPL 70/7116.

## 3 Results

### 3.1 Construction of a VACV strain WR lacking the innate immunomodulatory genes *N1L*, *C6L* and *K7R*

**Fig. 1:**
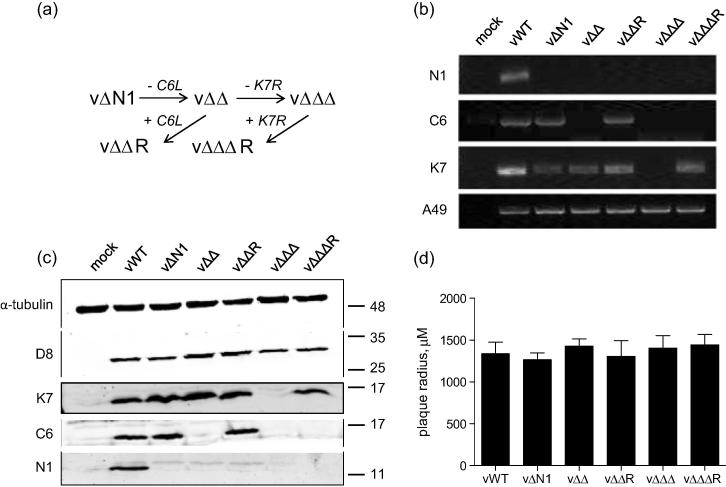
Construction of a VACV strain WR lacking three innate immunomodulators. (a) Schematic representation of the order in which the *C6L* and *K7R* genes were removed from a virus already lacking *N1L* (vΔN1). Revertant viruses were constructed at each stage as controls. (b) PCR analysis of proteinase K-treated lysates of BSC-1 cells infected with the indicated viruses for 16 h at 2 p.f.u. per cell using primers specific for *N1L*, *C6L*, *K7R* and *A49R*. (c) Immunoblot analysis of BSC-1 cells infected for 16 h with the indicated viruses at 2 p.f.u. per cell. Lysates were analysed by SDS-PAGE and immunoblotted using monoclonal antibodies against α-tubulin and D8 or with polyclonal antisera against N1, C6 and K7. Molecular mass markers are indicated on the right (kDa). (d) Monolayers of BSC-1 cells were infected with the indicated viruses for 72 h. Cells were stained with crystal violet and the size of 20 plaques was measured using Axiovision acquisition software and a Zeiss AxioVert.A1 inverted microscope. Results are expressed as the mean plaque radius ± SD. Data are representative of at least 2 independent experiments.

To construct a virus lacking *N1L*, *C6L* and *K7R*, *C6L* was deleted from a virus already lacking *N1L* (vΔN1, [17]) by transient dominant selection (see methods) yielding vΔΔ, followed by the removal of *K7R* yielding vΔΔΔ. As controls, a revertant virus where the deleted gene was re-inserted back into its natural locus was constructed at each stage (Fig. 1a). Deletion of *C6L* and *K7R* was confirmed by PCR analysis of proteinase K-treated lysates of infected BSC-1 cells using primers specific for these genes in addition to *N1L* and *A49R* as a control (Fig. 1b). The phenotype of the resulting recombinant viruses was confirmed at the protein level by immunoblotting of lysates from infected BSC-1 cells using antisera against N1, C6 and K7, as well as a monoclonal antibody against VACV protein D8 as an infection control (Fig. 1c). Proteins N1, K7 and C6 are each non-essential for virus replication or spread in cell culture [17], [18], [19] and measurement of the plaque size of mutants lacking 2 or 3 of these genes confirmed that deletion of these genes in combination did not affect viral spread in BSC-1 cells (Fig. 1d).

### 3.2 Deletion of *N1L*, *C6L* and *K7R* in combination attenuates the virus sequentially in murine intradermal and intranasal models of infection

**Fig. 2:**
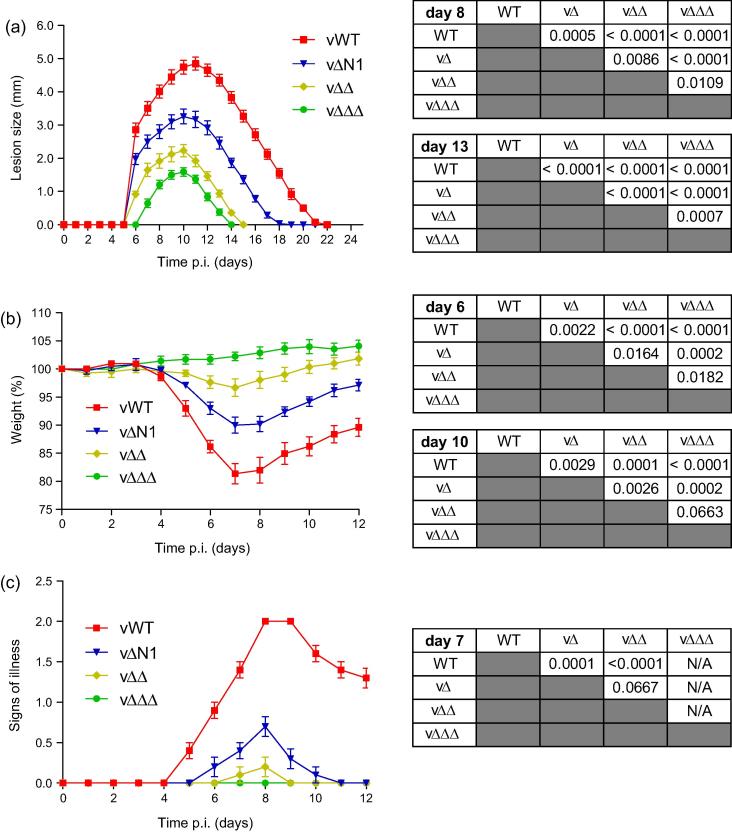
Sequential attenuation of recombinant viruses. (a) The lesions induced by i.d. inoculation of C57BL/6 mice (*n* = 5) with 10^4^ p.f.u. of the indicated viruses in both ear pinnae were measured daily. Data are expressed as the mean lesion size (mm) ± SEM. (b and c) BALB/c mice (*n* = 5) were infected i.n. with 5 × 10^3^ p.f.u. of the indicated viruses and their weights (b) and signs of illness (c) were monitored daily. Weight data (b) are expressed as the percentage ± SEM of the mean weight of the same group of animals on day 0. Signs of illness data (c) are expressed as the mean score ± SEM. Statistical analyses for data on the day p.i. indicated are in the tables on the right hand side and were determined using the Student’s *t*-test. N/A indicates that data for this comparison could not be determined. Data are representative of at least 2 independent experiments.

The *N1L* [17], [35], *C6L* [18] and *K7R* [19] single deletion viruses are attenuated in both the i.d. and i.n. models of murine infection, highlighting their importance as virulence factors. When compared side-by-side in the i.d. model, the level of attenuation was found to be similar amongst the three single deletion viruses (Suppl. Fig. 1a). In contrast, a virus lacking both N1 and C6 (vΔΔ) was significantly more attenuated than vΔN1, and a virus lacking all three immunodulators (vΔΔΔ) was attenuated further still (Fig. 2a), indicating that the roles of these innate inhibitors *in vivo* is non-redundant. The double deletion revertant (vΔΔR) and the triple gene deletion revertant (vΔΔΔR) control viruses behaved as expected, demonstrating that the observed attenuation phenotypes were due to specific genes deletions and not mutations elsewhere in the viral genome (Suppl. Fig. 1b). Similar results were obtained in the i.n. model of infection where deletion of C6 and K7 again led to sequential attenuation of the virus, as indicated by reduced weight loss (Fig. 2b) and fewer signs of illness (Fig. 2c).

### 3.3 Deletion of *N1L*, *C6L* and *K7R* in combination does not enhance vaccine potency

**Fig. 3:**
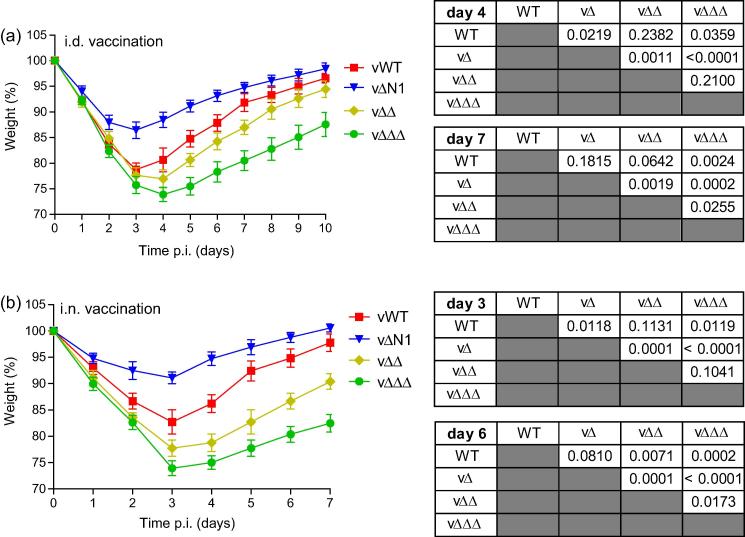
Vaccine potency of recombinant viruses. (a) Groups of five C57BL/6 mice were vaccinated by i.d. inoculation in both ear pinnae with 10^4^ p.f.u. of the indicated viruses and challenged i.n. 1 month later with 5 × 10^6^ p.f.u. of wild-type WR and the resulting weight change was monitored daily. (b) Groups of five BALB/c mice that were vaccinated i.n. with 5 × 10^3^ p.f.u. of the indicated viruses were challenged i.n. 6 weeks later with 5 × 10^6^ p.f.u. of wild-type VACV WR and the resulting weight change was monitored daily. Data are expressed as the percentage ± SEM of the mean weight of the same group of animals on day 0. Statistical analyses for data on the days p.i. indicated are in the tables on the right hand side and were performed using the Student’s *t*-test. All experiments were conducted at least twice and the data shown are representative.

To determine whether the double and triple gene deletion viruses have improved vaccine potency compared with the N1 single deletion virus, mice were vaccinated i.d. and challenged i.n. one month later with a lethal dose of wild-type VACV WR. As reported [10], single deletion of N1 provided the mice with better protection against challenge, indicated by significantly less weight loss over a period of 10 days (Fig. 3a). However, vΔΔ provided less protection than both vΔN1 and vWT (although this did not reach statistical significance when compared to the WT virus on any one day) and vΔΔΔ provided significantly less protection again (Fig. 3a). Similar results were observed following challenge of mice that were vaccinated i.n. (Fig. 3b). When compared head-to-head each of the single deletion viruses enhanced vaccine potency to a similar degree (Suppl. Fig. 2a) and the revertant control viruses behaved as expected in challenge experiments (Suppl. Fig. 2b).

### 3.4 The double and triple gene deletion viruses induce lower CTL responses

**Fig. 4:**
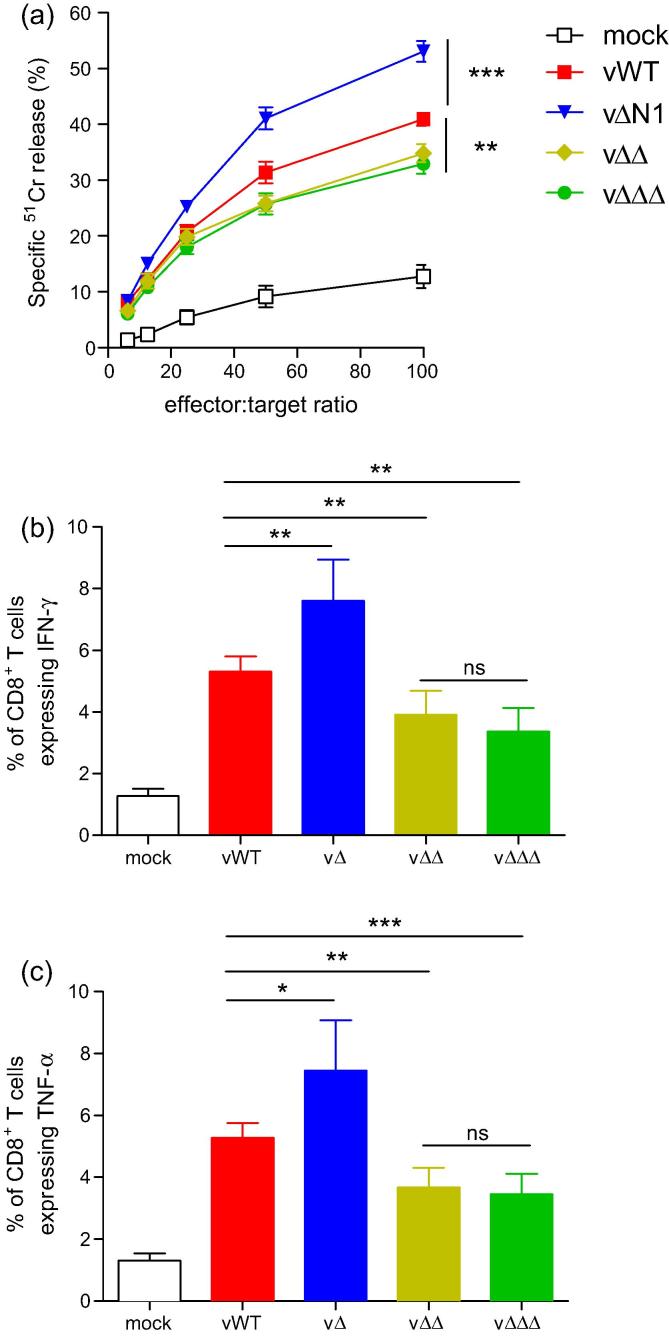
CD8^+^ T cells responses one month post-vaccination. The activity of splenic T cells from C57BL/6 mice (*n* = 5) was measured one month post-vaccination with 10^4^ p.f.u. of the indicated viruses, or mock-vaccination with PBS in both ear pinnae. (a) The cytolytic activity of splenic T-cells against VACV strain WR-infected EL-4 target cells was determined by ^51^Cr release assay. Data are expressed as percentage specific lysis ± SEM. (b and c) The percentage of CD8^+^ T cells expressing IFNγ (b) or TNFα (c) was determined by intracellular cytokine staining. Data are expressed as percentage ± SD. Statistical analyses were performed using the Student’s *t*-test. ^*^P < 0.05, ^**^P < 0.01, ^***^P < 0.001, ns: not significant. The experiments were conducted at least 3 times and the data shown are representative.

To understand why vaccination with vΔΔ or vΔΔΔ afforded less protection than vΔN1, CD8^+^ T cells responses one month post-i.d. vaccination were analysed. To measure the cytolytic activity of VACV-specific T cells a chromium release cytotoxicity assay was performed. The specific cytolytic activity of T cells from vΔN1-vaccinated mice was significantly higher than that from vWT-vaccinated animals (Fig. 4a), corroborating recently published findings [10]. Conversely, significantly lower VACV-specific cytolytic activity of T cells was measured in vΔΔ- or vΔΔΔ-vaccinated mice. Interestingly, no significant difference in cytolytic activity was observed between vΔΔ and vΔΔΔ.

The release of cytokines by splenic CD8^+^ T cells that were stimulated *ex vivo* with VACV peptides was also analysed by intracellular cytokine staining. In agreement with data published recently [10], splenic CD8^+^ T cells from vΔN1-vaccinated mice secreted enhanced levels of IFNγ (Fig. 4b) and TNFα (Fig. 4c) following stimulation than cells from vWT-vaccinated mice. In contrast, splenic CD8^+^ T cells from both vΔΔ- and vΔΔΔ-vaccinated mice secreted significantly less cytokines. Again, no significant difference was observed between the double and triple gene deletion viruses.

### 3.5 Double and triple gene deletion viruses induce lower neutralising antibody titres

**Fig. 5:**
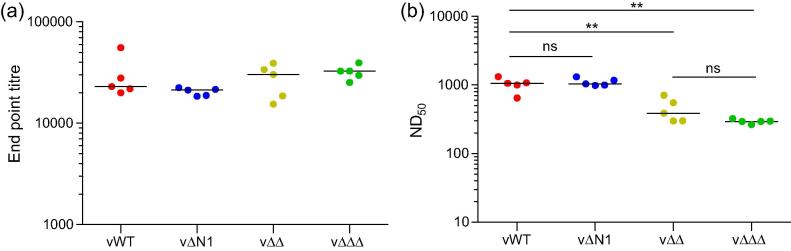
Humoral responses one month post-vaccination. Antibody responses of C57BL/6 mice (*n* = 5) were measured one month post-vaccination with 10^4^ p.f.u. of the indicated viruses in both ear pinnae. (a) Serum antibody end-point titres against VACV proteins were determined by ELISA. End-point titres were defined as the reciprocal serum dilution giving twice the optical density obtained from BSA. (b) The neutralisation capacity of antibodies in the serum was assessed by plaque-reduction neutralisation against VACV strain WR intracellular mature virus. The median value for each population is represented by a horizontal black bar. Statistical analyses were performed using the Student’s *t*-test. ^**^P < 0.01, ns: not significant. Experiments were conducted at least 3 times and representative data are shown.

The poorer immunogenicity of the double and triple gene deletion viruses might also be due to altered antibody responses. To investigate this possibility, sera were collected from mice one month post-i.d. vaccination and the VACV-specific antibody titres and VACV-specific neutralising titres were measured by ELISA and plaque reduction neutralisation respectively. The titre of VACV-specific antibodies measured by ELISA was not different between the groups of vaccinated mice and good antibody levels were induced in all cases (Fig. 5a). The same was not true, however, when the titre of neutralising serum antibodies was measured by plaque reduction neutralisation assay. In this case the ND_50_ of sera from both the vΔΔ- and vΔΔΔ-vaccinated mice was significantly lower than that from both vWT and vΔN1-vaccinated mice, although there was no significant difference between the double and triple gene deletion viruses (Fig. 5b).

## 4 Discussion

Inducing a robust innate immune response is an important step to designing an immunogenic vaccine and this is often achieved by the addition of a vaccine adjuvant. However, a full understanding of how the innate immune system impacts immune memory is lacking and would greatly enhance our ability to rationally design vaccines with enhanced immunogenicity profiles. VACV-based vectors are popular candidates, however their genomes still encode proteins with a known role in dampening the host innate immune response, which may negatively impact their potential use as vaccine vectors. Indeed, the deletion of numerous VACV immunomodulatory genes has been shown to enhance immunogenicity including the chemokine-binding protein A41 [21], [32], the IL-1β-binding protein B15 [21], [36], the inhibitor of MHC class II antigen presentation A35 [37], the IL-18-binding protein C12 [38], the type I (B18) and type II (B8) IFN-binding proteins [39], the IRF3/7 inhibitor C6 [11], [12], the NF-kB inhibitor and anti-apoptotic protein N1 [10], the dual NF-kB and IRF3/7 inhibitor K7 ([13] and Ren et al., unpublished results) and the TLR signalling inhibitor A46 [40]. Data presented here demonstrate that deletion of three of these genes (*N1L*, *C6L* and *K7R*) in combination from VACV WR did not further enhance the immunogenicity and in fact provided poorer protection than the single gene deletion viruses. These data highlight that in the context of a replicating vaccine vector there is a fine balance between viral attenuation and immunogenic potential.

Given that vΔΔ and vΔΔΔ are sequentially more attenuated than vΔN1 (Fig. 2) they may have generated lower antigen levels and/or been cleared more quickly by the host immune system and hence induced a weaker adaptive response. The importance of antigen availability for the formation of CD8^+^ T cell-dendritic cell interaction kinetics and the ensuing memory response was demonstrated recently [41]. Of further interest, the inferior CD8^+^ T cell responses and neutralising antibody titres observed with vΔΔ and vΔΔΔ were not significantly different between these two viruses, however vΔΔΔ provided worse protection than vΔΔ in the challenge studies (Fig. 3). These data may indicate that other aspects of immune memory such as CD4^+^ T cells, or the recently identified memory NK cells [42] may play an important role in determining vaccine efficacy of VACV. Whether equivalent protection to WT or vΔN1 vaccination could be achieved by increasing the vaccination dose of vΔΔ and vΔΔΔ warrants further investigation.

A recent study using an MVA vector expressing HIV-1 antigens (MVA-B) in the context of a DNA prime/MVA boost regime found that the deletion of *C6L* and *K7R* in combination enhanced the magnitude and quality of HIV-1-specific CD4^+^ and CD8^+^ T cell responses in mice, as well as Env antibody levels compared to the parental MVA-B vector and MVA-B lacking *C6L* alone [13]. These data demonstrate that in the context of a non-replicating strain of VACV, the deletion of more than one immunomodulatory gene may be beneficial. What is not clear from this study, however, is whether these memory responses would have been achieved with the deletion of *K7R* alone, because an MVA-B *K7R* single deletion was not included. Furthermore, whether these correlates of protection will translate to protection in an *in vivo* challenge model remains to be determined. Another recent study, also based on an MVA vector expressing HIV-1 antigens, found enhanced HIV-specific CD4^+^ and CD8^+^ T cell responses as well as Env-specific antibody responses in rhesus macaques with a vector lacking 4 immunomodulatory proteins [20]. However again this vector was not compared to single gene deletions and protection was not determined by a challenge experiment.

Mounting evidence indicates an important role for type I IFN [43], [44] in vaccine immunogenicity and each of the proteins selected for this study inhibits type I IFN production. Furthermore, the importance of NF-κB in generating robust memory immune responses was demonstrated recently for N1, an inhibitor of NF-κB and apoptosis. By vaccinating mice with viruses encoding N1 mutants that were competent for only one of these functions it was demonstrated that the anti-NF-κB activity of N1 was important for the enhanced protection observed with vΔN1, with no apparent contribution of its anti-apoptotic function [35]. These types of studies demonstrate how viruses lacking innate immunomodulators can be utilised as tools to further our understanding of the relationship between innate immunity and immune memory, which will be important for future vaccine design.

## Conflicts of interest

The authors declare no conflicts of interest.
