# Supplementary material for: Increased attenuation but decreased immunogenicity by deletion of multiple vaccinia virus immunomodulators
Source: Vaccine. 2016 Sep 14;34(40):4827–34. doi: 10.1016/j.vaccine.2016.08.002 (PMC5022402; doi:10.1016/j.vaccine.2016.08.002)
Supplement: Supplementary figures [file mmc1.pptx]

## Slide 1
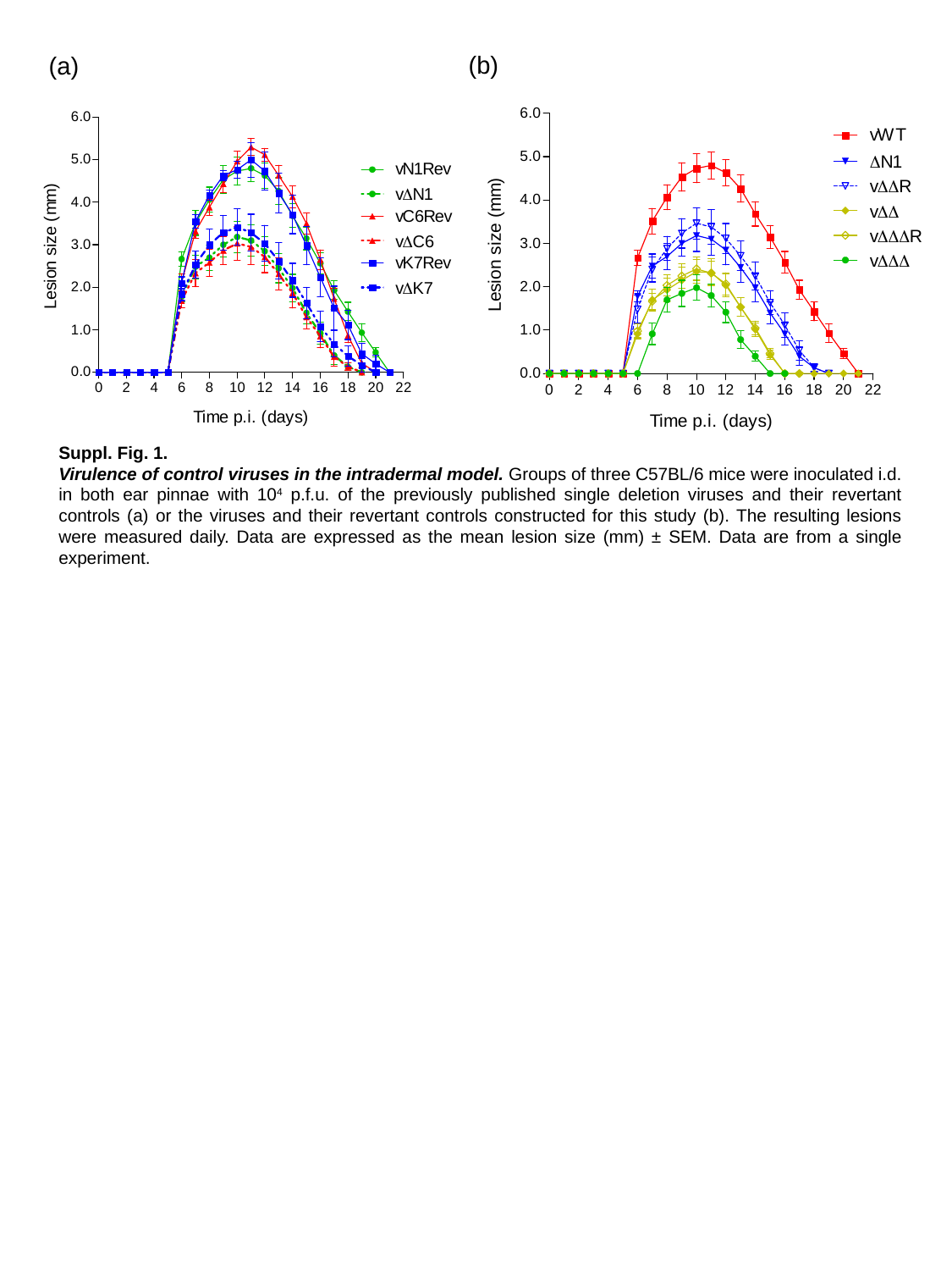

(b)
(a)
Suppl. Fig. 1.
Virulence of control viruses in the intradermal model. Groups of three C57BL/6 mice were inoculated i.d. in both ear pinnae with 104 p.f.u. of the previously published single deletion viruses and their revertant controls (a) or the viruses and their revertant controls constructed for this study (b). The resulting lesions were measured daily. Data are expressed as the mean lesion size (mm) ± SEM. Data are from a single experiment.

## Slide 2
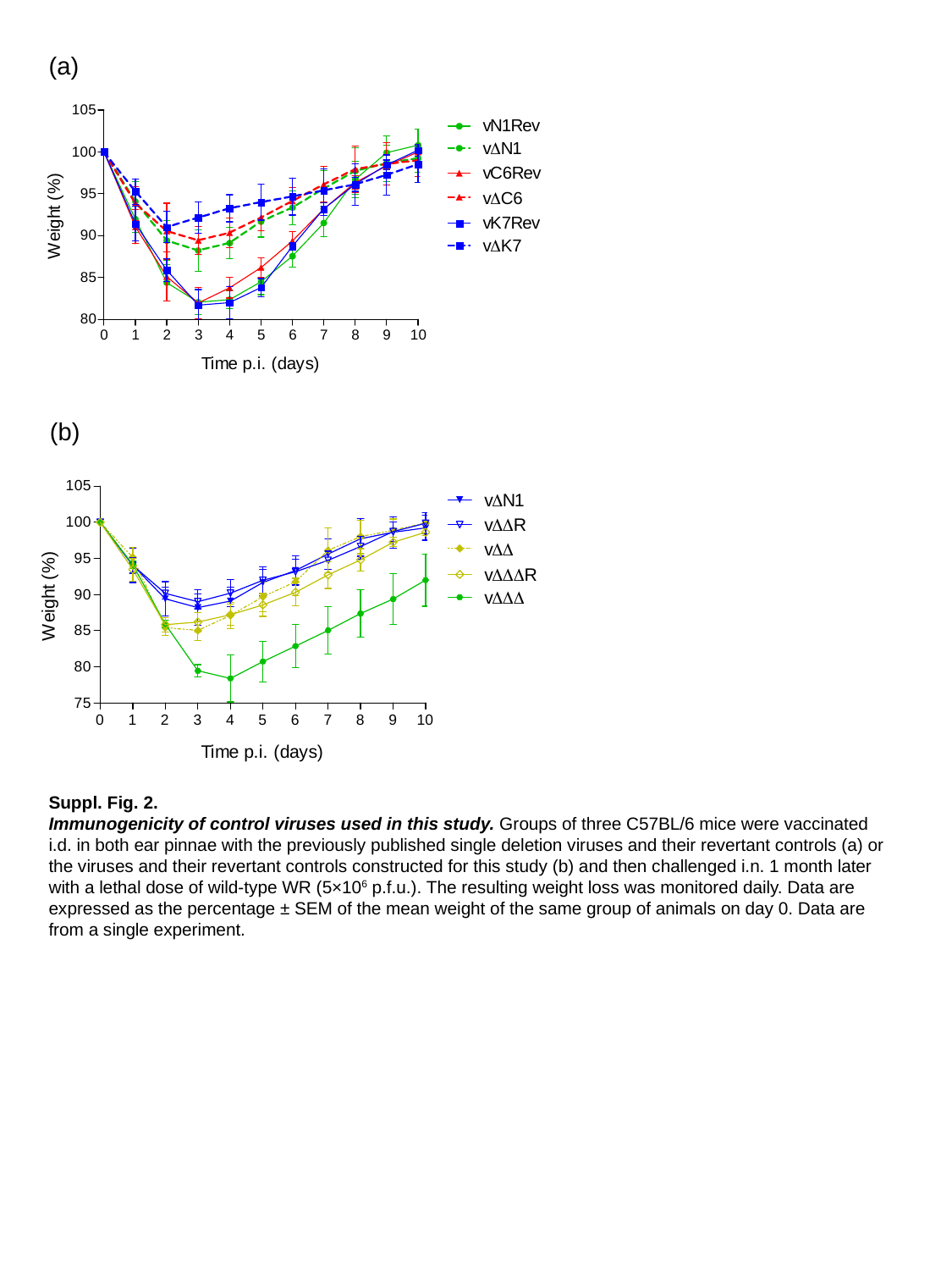

(a)
(b)
Suppl. Fig. 2.
Immunogenicity of control viruses used in this study. Groups of three C57BL/6 mice were vaccinated i.d. in both ear pinnae with the previously published single deletion viruses and their revertant controls (a) or the viruses and their revertant controls constructed for this study (b) and then challenged i.n. 1 month later with a lethal dose of wild-type WR (5×10^6^ p.f.u.). The resulting weight loss was monitored daily. Data are expressed as the percentage ± SEM of the mean weight of the same group of animals on day 0. Data are from a single experiment.
